# Effect of an educational intervention on breastfeeding knowledge and attitude among interns at Cairo University Hospital

**DOI:** 10.1186/s42506-019-0020-y

**Published:** 2019-06-13

**Authors:** Ola A. Mostafa, Marwa R. Salem, Ahmed M. Badr

**Affiliations:** 1 Department of Public Health and Community Medicine, Faculty of Medicine, Cairo University, PO Box: 109, El Malek El Saleh, Cairo, 11559 Egypt; 2 Department of Pediatrics, Faculty of Medicine, Cairo University, PO Box: 109, El Malek El Saleh, Cairo, 11559 Egypt

**Keywords:** Breastfeeding, Interns, Education, Knowledge, Attitude, Interventional study

## Abstract

**Background:**

Professional advice provided to mothers has an effective role on the prevalence and duration of breastfeeding. Previous studies showed that health care providers had defective knowledge and skills necessary to promote and support breastfeeding.

**Aim:**

To assess breastfeeding-related knowledge and attitude among interns at Cairo University Hospital, before and after the provision of breastfeeding educational training sessions.

**Materials and methods:**

The first phase was a cross-sectional study, conducted in Cairo University Hospital (Kasr Al Ainy) among 137 interns. The second phase was a pre-post interventional design. A pretested self-administered questionnaire was used to explore breastfeeding-related knowledge and attitude before, immediately after, and 3 months after breastfeeding educational sessions.

**Results:**

Participants’ mean age was 23.7 ± 0.81, (range 22–27 years), with equally distributed males and females. The median total knowledge percent score was 56.4 (45.2–64.5). The highest median subtotal knowledge percent score was for effective feeding 100 (100–100), and the least median was for breast milk expression 20 (0:40). Participants’ knowledge improved after the educational intervention: The subtotal knowledge scores showed a statistically significant improvement immediately after and 3 months after the intervention in the following items: advantages for the baby, colostrum, duration, complementary feeding, and breast milk expression. The median total attitude percent score was 80 (74.1–83.5) and significantly improved immediately after the intervention.

**Conclusion:**

Baseline knowledge and attitude scores among interns significantly improved after the intervention. Therefore, adoption of different curricular and extracurricular activities to improve breastfeeding knowledge and skills is required.

## Introduction

The benefits of breastfeeding for mothers and infants have been well documented, and new evidence about breastfeeding benefits continues to emerge from the scientific community [1]. However, the data of UNICEF, the World Health Organization(WHO), and the World Bank (2016) showed that 40% of the world’s infants under 6 months of age were exclusively breastfed, continued breastfeeding rates have dropped to 46% at 2 years, and about one third of infants 6–8 months old were not eating solid foods [2].In Egypt, breastfeeding practices are not always optimal. Data from the Egyptian Demographic and Health survey [3] showed that six in ten children were reported to have received a prelacteal feed after birth, and only four in ten children under 6 months of age were being exclusively breastfed. Less than one quarter of children aged 6–23 months were being fed according to minimum infant and young child feeding standards for diet diversity and meal frequency [3].

The professional advice given to mothers in health facilities impacts all steps of infant feeding [4]. However, studies conducted to assess the knowledge, attitude, and practice of breastfeeding among healthcare providers showed that they had inadequate knowledge, attitude, and skills necessary to promote breastfeeding and complementary feeding practices [5].The main reasons for such an existing gap in knowledge and skills are inadequate coverage of breastfeeding and weaning in medical curricula as well as defective in-service updates [6].

Though breast-feeding promotion has been a high priority in Egypt for several decades in the in-service continuing education program of the Ministry of Health and Population (MOHP) [7] and was also part of the IMCI training [8], few studies have tested the effectiveness of breastfeeding educational interventions for the health professional and interns [9]. The current study was conducted to explore the baseline knowledge and attitudes towards breastfeeding among interns, at Cairo University Hospital and measure the effect of a breastfeeding educational intervention on their breastfeeding-related knowledge and attitude.

## Materials and methods

### Setting

The researchers conducted the current study at the hospital affiliated to the Faculty of Medicine, Cairo University.

### Study design and phases

Phase I was an exploratory cross-sectional study to assess the breastfeeding-related knowledge and attitude among participating interns, as well as their willingness and suggestions to improve their knowledge and skills in the field of breastfeeding.

Phase II was an interventional design to measure the effect of training sessions on breastfeeding-related knowledge and attitude among medical interns.

The study took place over a 6-month duration.

### Population and sampling method

#### Population

Medical interns in the academic year 2017/2018 were selected for the study because this is a phase where they would have already been exposed to the various aspects of basic and clinical subjects which address the needs, advantages, and status of infant-feeding practices. Thus, exploring their current knowledge on infant feeding practices and providing them with adequately designed health education sessions about breastfeeding would help them in better interaction with expectant and lactating mothers once they start their full-time clinical practice. During their internship, interns are distributed over six groups for rotations in different specialties. A purposive sample was used to select the first group of interns who already finished their rotations in the Obstetrics and Gynecology as well as the Pediatrics departments, where interns could have been exposed to breastfeeding education. This selection also provided enough time for the researchers to assess, intervene, and evaluate the immediate and 6 months after the intervention on intern’s breastfeeding knowledge and attitude.

#### Sample size

Among the selected interns who fulfilled the previous inclusion criteria (*n* = 150), 146 agreed to participate (response rate 97.3%) and fill in the questionnaire, but nine were excluded as the questionnaires were not filled in correctly. This condition resulted in including 137 interns in the study. For the interventional phase, using MedCalc program version 11.0 and mean difference in knowledge percent score 11 ± 8.95 [10] with alpha = 0.05 and power 99%, the calculated sample size was found to be 15; adding a 20% expected non-response rate, the sample size was 18 interns. Fifty interns were recruited to the study. Those 50 interns were selected from the total 137 by a simple random sample to be included in this phase of the study. Six were dropped out, and only 44 were included (a response rate of 88%) as they attended the two educational days and filled in the required questionnaires (before, immediately after, and 3 months after the educational intervention).

#### Intervention

Medical interns were invited to attend two educational sessions, 2 h duration each. The sessions were carried out by the IBCLC (International Board Certified Lactation Consultant) over 2 days. They covered all components of the 20-h UNICEF/WHO course for breastfeeding promotion and support in a baby-friendly hospital [11].These components are updated technical information and additional clinical practice sessions (using dolls and role plays) and an emphasis on supportive practices during labor and birth, skin-to-skin contact, early initiation of breastfeeding, recommendations for breastfeeding duration, breastfeeding problems management, preparation for discharge, and HIV considerations. The current study’s intervention covered briefly all the items of the 20-h course in the form of a power point presentation, discussion, and problem-solving. Interns were asked to fill in a questionnaire before sessions, immediately after the end of the last session, and 3 months later.

### Data collection

A pre-tested self-administered questionnaire was used and included the following items:

1. Personal data including age, sex, nationality, and planned specialty
2. Knowledge about breastfeeding including the following questions: advantages for the baby, maternal benefits versus limitations, colostrum, adequate feeding, duration of feeding, complementary feeding, breast milk expression, problems with breastfeeding, and the practical aspect of breastfeeding. The researchers constructed the knowledge questions based on the content of the UNICEF/WHO 20-h course [11]. A sixty-one closed-ended format was used with true/false and do not know options. A score of 1 was assigned to the correct answer, and a score of 0 was assigned to the incorrect or do not know answers. The total score ranged from 0 to 61. The number of correct answers was added for each item to obtain nine subtotal knowledge scores, and a total score was calculated to measure the total knowledge score by adding the number of correct answers for the nine items. Transforming the subtotal and total raw scores into percent scores was done by dividing the raw score for each item on the maximum achievable score and then multiplying the result by 100
3. Attitude towards breastfeeding was tested using the following tools:

- Iowa Infant Feeding Attitude Scale (IIFAS), a validated instrument shown to have a Cronbach *α* ranging from 0.85–0.86 [12] .The IIFAS covers various dimensions of infant feeding within its 17 statements, and respondents were asked to indicate the extent to which they agree with each statement on a five-point Likert scale ranging from 1 = “strongly disagree” to 5 = “strongly agree.” IIFAS scores range from 17 to 85, with a higher score indicating a more positive attitude towards breastfeeding. Transforming the total raw score into percent score was done by dividing the raw score on the maximum achievable score and then multiplying the result by 100.
- For further assessment of interns’ attitude towards breastfeeding in practice:

I. All interns were asked if they would encourage expectant and lactating mothers who are their relatives or friends to breastfeed.
II. Female interns were asked if they would breastfeed their babies as future mothers.
III. Male interns were asked if they would encourage their future wives to breastfeed their babies.
IV. All interns were asked about their opinion towards incorporation of a mandatory breastfeeding course during the internship year, and about their willingness to participate in breastfeeding clinic activities.

4. Interns’ recommendations to improve breastfeeding education: the provided recommendations were recorded, grouped and presented as percentages.

The questionnaire was pretested with a convenience sample of 10 interns (not included in the study), and the required modifications were done.

### Statistical analysis

Pre-coded data were entered into the Statistical Package of Social Science (SPSS) version 21. The data were summarized using mean and standard deviation for quantitative normally distributed variables, while the median and interquartile range (IQR) were used for quantitative variables, which were not normally distributed. Numbers and percentages were used for qualitative variables. Comparisons between groups were done using the Chi-square test for qualitative variables, non-parametrical Mann-Whitney *U* test and Kruskul-Wallis tests for quantitative variables which were not normally distributed.

Testing the effect of breastfeeding education on knowledge and attitude scores was done using non-parametrical Freidman test and Wilcoxon sign rank test for quantitative variables which were not normally distributed. For qualitative data, Cochran’s Q and McNemar tests were used. Nonparametric correlations were done to test the relation of interns’ age with their knowledge and attitude scores. *P* values less than or equal to 0.05 were considered statistically significant.

## Results

One hundred and thirty seven interns participated in the cross-section phase, while 44 interns participated in the intervention phase. Table 1 shows the background characteristics of the participants: Their mean age was 23.7 ± 0.81, ranging from 22 to 27 years. Half (50.4%) of them were females. The majority (97.8%) were Egyptians. The highest reported planned specialty was pediatrics (24%). Only 8.8% of the participants had a previous training course in breastfeeding. The majority (81.0%) described the scientific material offered during the undergraduate years as useful.

**Table 1:** Background characteristics of the participating interns (*n* = 137) at Cairo University Hospital, Egypt, 2017–2018

Variable	Value
Age mean ± SD	23.7 ± 0.81
Sex No. (%)	
Male	68 (49.6)
Female	69 (50.4)
Nationality	
Egyptian	134 (97.8)
Non Egyptian	3 (2.2)
Had previous training courses in breastfeeding	
Yes	12 (8.8)
No	125 (91.2)
Opinion about undergraduate breastfeeding education	
Useful	111 (81)
Not useful	26 (19)

As shown in Table 2, among the subtotal knowledge percent scores, the highest median was for effective feeding (100), followed by advantages for the baby and maternal benefits (83.3 each). The least median was for breast milk expression (20) followed by the median for problems with breastfeeding (40). The median attitude percent score was 80, while the median total knowledge percent score was 56.5.Testing the relation between breastfeeding total knowledge and attitude percent score and personal data of interns showed that no association was found with interns’ age, sex, planned specialty, or nationality.

**Table 2:** Baseline percent scores of breastfeeding knowledge and attitude among interns (*n* = 137) at Cairo University Hospital, Egypt, 2017–2018

Item percent score Knowledge:	Mean ± SD	Median (Q1:Q3)
Advantages for the baby	76.2 ± 19.5	83.3 (66.7:83.3)
Maternal benefits versus limitations	74.9 ± 16.7	83.3 (66.7:83.3)
Colostrum	72.2 ± 25.7	75.00 (50:100)
Effective feeding	93.1 ± 16.5	100 (100:100)
Duration	55.69 ± 25.75	58.3 (33.3:66.7)
Complementary feeding	56.8 ± 24.9	60 (40:80)
Breast milk expression	24.4 ± 24.9	20 (0:40)
Problems with breastfeeding	42.8 ± 15.4	40(28.3:46.7)
Practical aspects of breastfeeding	50.1 ± 18.7	50 (33.3:66.7)
Total knowledge	55.8 ± 11.9	56.45 (45.2: 64.5)
Attitude towards breastfeeding	78.8 ± 8.1	80 (74.1: 83.5)

A statistically significant improvement in interns’ subtotal knowledge scores from baseline to immediately after, and 3 months after the intervention was observed in the following items: advantages for the baby, colostrum, duration, complementary feeding, and breast milk expression. However, the improvement was noticed between immediately after and 3 months after the education was not statistically significant as depicted in Table 3.

**Table 3:** Interns’ (*n* = 44) knowledge and attitude percent scores before and after the educational breastfeeding intervention at Cairo University Hospital, 2017–2018

Knowledge scores	Before intervention	Immediately after intervention	3 months after intervention	*P* values (Wilcoxon sign rank test)		
	Mean ± SD Median (IQR)	Mean ± SD Median (IQR)	Mean ± SD Median (IQR)	P1a	P2b	P3c
Advantages for the baby	71.2 ± 21.7 83.3 (66.7–83.3)	93.6 ± 14.0 100 (100–100)	95.8 ± 7.3100 (87.5–100)	< 0.001*	0.13	< 0.001*
Maternal benefits versus limitations	75 ± 16.3 75 (66.7–83.3)	93.6 ± 9.7 100 (83.3–100)	87.1 ± 16.0 83.3 (83.3–100)	< 0.001*	0.025	0.001*
Colostrum	68.8 ± 24.2 75 (50–75)	89.8 ± 16.5 100 (75–100)	86.9 ± 19 100 (75–100)	< 0.001*	0.30	0.001*
Effective feeding	90.9 ± 18.1 100 (100–100)	92.4 ± 17.4 100 (100–100)	92.4 ± 15.9 100 (100–100)	0.64	0 .73	0.79
Duration	55.7 ± 27.4 58.3 (33.3–83.3)	88.3 ± 15.1 91.7 (83.3–100)	85.2 ± 17 91.7 (70.9–100)	< 0.001*	=.098	< 0.001*
Complementary feeding	56.4 ± 26 60 (40–80)	76.4 ± 23.3 80 (60–100)	70. ± 24.6 80 (60–100)	< 0.001*	0.15	0.005*
Breast milk expression	21.9 ± 24.7 20 (0–40)	46.8 ± 22 40 (40–60)	48.6 ± 22.6 40 (40–60)	< 0.001*	0.67	< 0.001*
Problems with breastfeeding	39.1 ± 14.6 40 (28.3–46.7)	82.9 ± 14.5 86.7 (73.3–93.3)	67.1 ± 18.9 73.33 (55–80)	< 0.001*	< 0.001	< 0.001*
Practical aspects of breastfeeding	47.4 ± 20.5 50 (27–66.7)	74.1 ± 14.5 75 (66.7–83.3)	65.3 ± 19.9 66.7 (50–83.3)	< 0.001*	0.003	< 0.001*
Total knowledge	53.3 ± 12.4 52.4 (45.2–62.5)	81.2 ± 8.6 80.7 (74.6–88.7)	74.5 ± 11.5 75.8 (69.4–81.9)	< 0.001*	< 0.001	< 0.001*
Attitude	80.2 ± 8.6 81.2 (75.6–84.4)	86.6 ± 6.6 87.1 (82.4–91.8)	83.4 ± 9 84.71 (77.9–89.4)	< 0.001*	.027	.005*

^a^P1: *p* value between before and immediately after intervention

^b^P2 *p* value between immediately after and 3 months after intervention

^c^P3 *p* value between before and 3 months after intervention

**Fig. 1:**
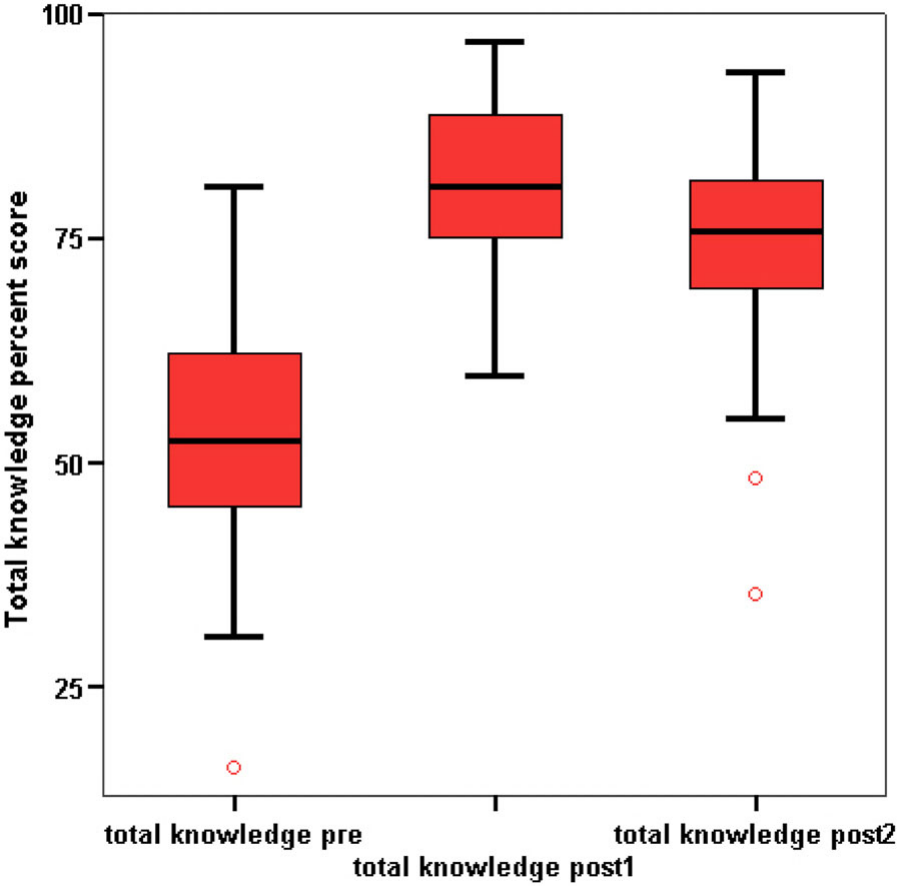
Median total knowledge percent score before and after the education sessions

**Fig. 2:**
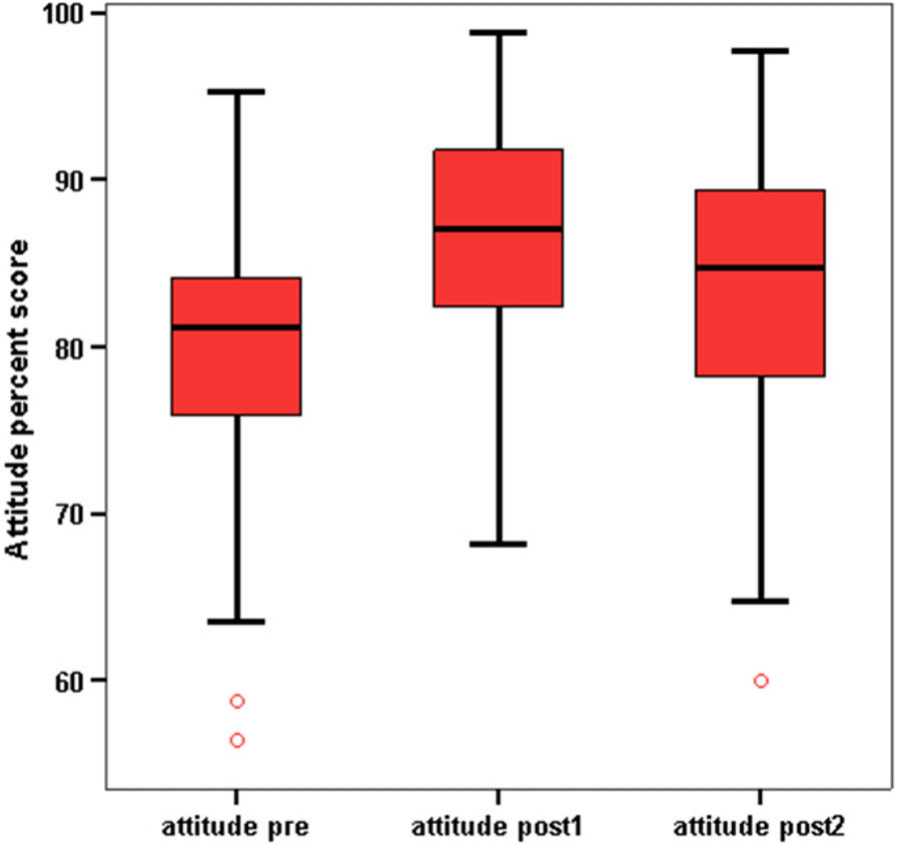
Median attitude percent score before and after the education sessions

There was a significant improvement in interns’ knowledge in the following items: maternal benefits versus limitations, problems with breastfeeding, practical aspects of breastfeeding, total knowledge score (as demonstrated in Fig. 1), and total attitude percent score (as shown in Fig. 2) between baseline, immediately after, and 3 months after the intervention. However, there was a significant decline in knowledge score between immediately after to 3 months after the intervention.

No significant change was found between baseline, immediately after, and 3 months after the intervention in effective feeding and subtotal knowledge scores. The percentages of interns who would encourage expectant and lactating mothers who are their relatives or friends to breastfeed were 95.5%, 95.5%, and 97.7% at baseline, immediately after, and 3 months after the intervention, respectively, with no statistically significant differences.

Among the participating female interns, the percentages of those who would intend to breastfeed their babies when they become mothers were as follows 100%, 95.5%, and 90.9% at baseline, immediately after, and 3 months after the intervention, respectively, with no statistically significant differences. Similarly, among participating male interns, the majority (96.8%) reported that they would encourage their wives to breastfeed their babies as future fathers at baseline, immediately after, and 3 months after the intervention with no statistically significant differences.

**Fig. 3:**
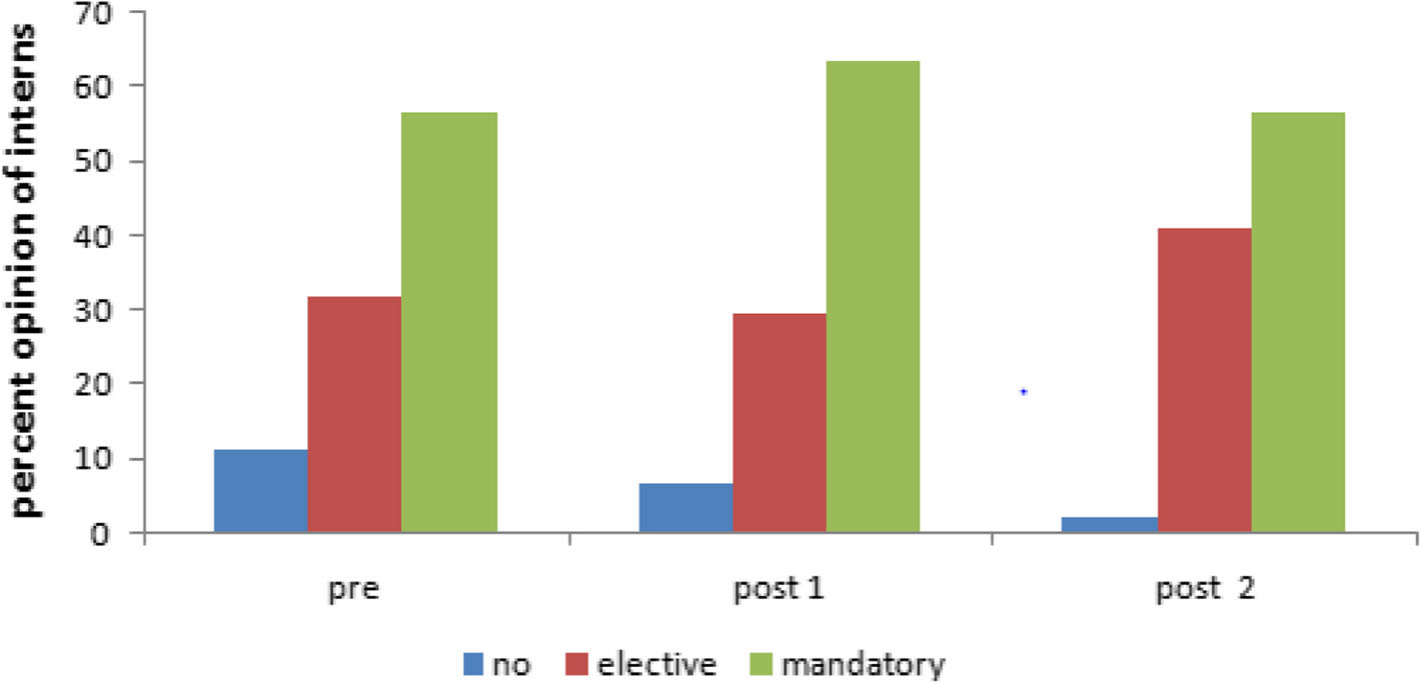
Percent opinion about having educational breastfeeding course during the internship year before and after the education sessions

The percentage of interns who agreed to receive a breastfeeding course during the internship year was 88.6% at baseline (56.8% mandatory versus 31.8% elective), while immediately after education, 93.1% agreed to have the course (63.6% mandatory versus 29.5% elective). Three months after the intervention, 97.7% agreed to have the course (56.8% mandatory versus 40.9% elective) with no statistically significant differences (Fig. 3).

## Discussion

The current study findings showed an improvement of level of knowledge and attitude of the interns towards breastfeeding after the educational intervention. These results are consistent with previous studies conducted in Croatia [13] and in developing countries [14]. In the present study, the median attitude percent score was 80. This finding was in accordance with that revealed from the survey conducted by Amin and colleagues among medical students in Saudi Arabia [15]. This finding might be explained by the fact that in the Middle East countries which are mainly Islamic, the Islamic culture is very supportive of breastfeeding (BF) [16].The Qur’an promotes breastfeeding: “Mothers shall give suck to their children for two full years for those who desire to complete the term” (Qur’an, 2:233). In contrast to the results of the current study, Singh [17] who assessed the attitudes of 2,500 urban college females in India and found that a majority of participants held negative attitudes towards BF, and Ahmed and El-Gindy in Egypt where the neutral attitude towards BF was dominant among undergraduate nursing students [5]. Research has demonstrated that support and positive attitude towards breastfeeding by medical practitioners gave positive messages to mothers that helped to establish and then maintain breastfeeding [4].

Although the current study indicated that interns had a high baseline score for median attitudes (80) towards BF, it revealed low median total knowledge percent score especially for the problems with breastfeeding- and milk expression-related knowledge. This finding was in accordance with those disclosed from different studies conducted among medical students, where significant knowledge deficit was revealed [18, 19]. This result might be explained by the fact that training for doctors about BF is often described as inadequate [13], with many relying on personal BF experience as the primary source of BF knowledge and skill development [18, 20]. Physicians are believed to play an essential role in promoting BF to women. BF initiation and duration increase when doctors give information, support, and encouragement to women [18].Therefore, it is essential for medical students to have the correct knowledge so that they can educate the public in the future.

In contrast to the current study that revealed no statistically significant difference between males and females about their breastfeeding-related knowledge and attitude, a survey conducted in the USA by Kavanagh et al. [19] reported that female participants had much higher breastfeeding knowledge scores than their male counterparts. Marrone et al. [21] also reported the same finding in the USA. This finding might be explained by the fact that both male and female medical students were exposed to the same breastfeeding-related information through their clinical rounds and lectures.

Despite the unacceptable level of breastfeeding knowledge among the interns, the majority of them thought that breastfeeding training in their undergraduate curricula was useful. This might reflect the “fragmented” nature of the undergraduate medical education where knowledge is introduced in bits and pieces without providing the skills for its practical application.

The current study findings confirmed that an interactive educational intervention about breastfeeding resulted in improved knowledge of interns. Previous study findings revealed that small, interactive workshops could increase the awareness of general practitioners about breastfeeding [22]. The conclusion of the current study coincides with other reviewed studies in other countries which were conducted using a pre-post intervention designing where health workers’ nutrition and breastfeeding knowledge increased after nutrition training like Italy [23], USA [10], Croatia [13], and other developing countries such as India, Pakistan, West Africa, and Malawi [14].

### Limitations of the study

The current study assessed knowledge and attitude of interns towards breastfeeding. Translating this knowledge into practice in terms of consultation of expectant and breastfeeding mothers had not been assessed. The justification is that interns are not yet authorized to offer patient consultation before getting their license of medical practice at the end of the internship year. Another limitation is that the study findings apply only to Cairo University interns. Such a finding cannot be generalized to other health care facilities.

## Conclusion and recommendations

The current study findings highlighted defective interns’ knowledge about breastfeeding and the improvement in their knowledge and attitude after the educational intervention. This finding reflects the need to adopt different curricular and extracurricular activities necessary to improve breastfeeding knowledge and skills required for a competent physician including content analysis of undergraduate breastfeeding education curriculum and provision of mandatory and elective breastfeeding courses under the supervision of trained faculty staff. Further studies are recommended to assess their breastfeeding consultation practice after the internship year.

## Data Availability

The datasets used and/or analyzed during the current study are available from the corresponding author on reasonable request.
